# The Genus *Curcuma* and Inflammation: Overview of the Pharmacological Perspectives

**DOI:** 10.3390/plants10010063

**Published:** 2020-12-30

**Authors:** Md. Moshiur Rahaman, Ahmed Rakib, Saikat Mitra, Abu Montakim Tareq, Talha Bin Emran, A. F. M. Shahid-Ud-Daula, Mohammad Nurul Amin, Jesus Simal-Gandara

**Affiliations:** 1 Department of Pharmacy, Noakhali Science and Technology University, Noakhali 3814, Bangladesh; raihannstu711@gmail.com; 2 Department of Pharmacy, Faculty of Biological Sciences, University of Chittagong, Chittagong 4331, Bangladesh; rakib.pharmacy.cu@gmail.com; 3 Department of Pharmacy, Faculty of Pharmacy, University of Dhaka, Dhaka 1100, Bangladesh; saikatmitradu@gmail.com; 4 Department of Pharmacy, International Islamic University Chittagong, Chittagong 4318, Bangladesh; montakim0.abu@gmail.com; 5 Department of Pharmacy, BGC Trust University Bangladesh, Chittagong 4381, Bangladesh; talhabmb@bgctub.ac.bd; 6 Department of Pharmacy, Atish Dipankar University of Science and Technology, Dhaka 1230, Bangladesh; 7 Pratyasha Health Biomedical Research Center, Dhaka 1230, Bangladesh; 8 Nutrition and Bromatology Group, Department of Analytical and Food Chemistry, Faculty of Food Science and Technology, University of Vigo–Ourense Campus, E32004 Ourense, Spain

**Keywords:** *Curcuma*, inflammation, pro-inflammatory cytokines, anti-inflammatory agents, pharmacology

## Abstract

The *Curcuma* genus has been extensively used for therapeutic purposes in traditional or folk medicine worldwide, including for its anti-inflammatory activity. *Curcuma* spp.’s active constituents, such as alkaloids, flavonoids, and terpenoids, can act on various targets in the signaling pathway, restrain pro-inflammatory enzymes, lower the production of inflammatory cytokines and chemokines, and reduce oxidative stress, which subsequently suppresses inflammatory processes. Preclinical and clinical studies have reported the predominant anti-inflammatory activity of several *Curcuma* species. This review provides an overview of the anti-inflammatory effects of different extracts, preparations, and bioactive components in this genus. This analysis may provide a scientific basis for developing new and alternative methods for the isolation of a single entity from this genus to attenuate inflammatory conditions. The *Curcuma* genus is waiting for researchers interested in developing safe and efficient anti-inflammatory agents for further investigation.

## 1. Introduction

Humans’ search for drugs from natural sources dates from ancient times and there is abundant evidence from various sources, such as written documents, preserved monuments, and even original plant medicines. People have been fighting against several diseases for many years, resulting in creating an awareness of medicinal plant usage. Thus, they learned to pursue drugs from various plant parts, including barks, seeds, fruit bodies, as even the whole plant [1].

The history of medicinal plants dictates how important they are in healing several complications and discovering new drug molecules in the modern era. Various examples describe the importance of medicinal plants as drug sources, such as aspirin, which has been used for millennia to treat pain and fever, where salicylic acid is the active metabolite of aspirin that was extracted from the bark of willow trees [2]. The Ebers Papyrus (c.1550 BC) from ancient Egypt demonstrated the use of *Cannabis sativa* applied topically for inflammation [3]. Digoxin, a cardiac glycoside that was extracted from the *Digitalis purpurea* plant was important for healing heart conditions long before the discovery of the glycoside [4]. In every period, during every successive century from the development of humankind to advanced civilizations, the healing properties of certain medicinal plants have been identified, noted, and conveyed to the successive generations. This perpetual interest in medicinal plants for providing drug molecules has brought about today’s modern and sophisticated fashion of their processing and usage as lead compounds for drug discovery [1].

Inflammation is regarded as a complex biological process that is mainly mediated by the disruption of tissue homeostasis. Inflammation is triggered by the presence of different biological, chemical, or physical agents and can be defined as acute or chronic depending on the type of stimuli. The immune system is always active to eliminate the damaging effect of acute inflammation; however, the failure of this response paves the way for a chronic phase, leading to an inflammatory cascade, which can ultimately develop several diseases, including chronic asthma, rheumatoid arthritis, inflammatory bowel disease, and psoriasis. Experimental studies and clinical epidemiological data have established chronic infections and inflammation as significant risk factors for different forms of cancer [5]. In the past, nearly 15–20% of all cancer deaths have been estimated as being due to underlying infections and inflammatory reactions [5]. Although several therapeutic agents, including corticosteroids, non-steroidal anti-inflammatory drugs (NSAIDs), and biologics drugs, are used mainly in the case of inflammation, they possess not only various adverse effects but these biologics are expensive. Therefore, to limit the drawbacks of both synthetic and biologic drugs, phytocompounds obtained from numerous medicinal plants have been used as ideal substitutes. The enormous research work on medicinal plants consolidates the hypothesis for using plant extracts as therapeutic agents due to the synergistic and additive effects of individual constituents [5].

In this review, we summarize the anti-inflammatory properties of the *Curcuma* genus, which could serve as the basis for developing new and alternative approaches for preventing and treating inflammatory conditions. Additionally, we presented the bioactive compounds, clinical, preclinical studies, and commercial perspectives of the *Curcuma* genus.

## 2. The Genus *Curcuma*

The *Curcuma* genus is a rhizomatous annual or perennial herb in the Zingiberaceae family. Almost all of the 120 species of this genus have already been included in many works of literature [6]. Most of the species of *Curcuma* are naturally present in tropical evergreen areas. Malaysia, Indochina, northern Australia, Thailand, India, etc. are the most favorable places to grow *Curcuma* species [7,8,9,10]. The chromosome number of these genus ranges from 2*n* = 20 to 105 [11]. The morphological features of various species in the *Curcuma* genus were examined by several authors [12,13,14,15,16]. When we analyzed the anatomy, *Curcuma* species differ considerably in terms of taxonomic characteristics. Four tribes of the Zingiberaceae family, namely, Zingibereae, Hedychieae, Globbeae, and Alpinieae, have received attention on the basis of morphologic traits, such as the locules count and the location of ovaries, alterations of the productive anther, staminodes development, and the orientation of rhizome shooting leaves in which *Curcuma* forms part of Zingibereae [6]. *Curcuma*’s rhizomes are sometimes considered to be fleshy, aromatic, and branched. Rhizomes become stuck with roots that often contain ellipsoid or conical tubers [12]. Basal leaf blades are typically lanceolate, broad, or rectangular in form, and are seldom linear [13]. To recognize the *Curcuma* species, flowers form single flexible anthers and spiral bracts with large compound spike inflorescences are prominent characteristics. The marginal bracts are indeed long and sometimes vividly colored and form a sterile cluster [14]. It has two separate flowering cycles. Prior to the growth of leafy shoots, short flowering plants produce flowers laterally from rhizomes and delayed flowering species typically develop terminally from the leafy shoots [15]. The heights of the plants are approximately between 50 and 200 cm. The species of *Curcuma* are mainly triploid and asexually replicate via rhizomes; they are incapable of developing seeds [16].

*Curcuma* has several application areas and is a commercially valuable genus. The species of this genus can contribute economically due to possessing preservative, medicinal, and flavoring properties [17,18]. Furthermore, significant sources of yellow dye are from the underground rhizome of *Curcuma* [19]. The Arabian word “kurkum”, which means yellow, was derived from the term *Curcuma* [20]. A lot of species of this genus possess a considerable amount of therapeutic potency by which numerous health disorders can be managed, including those related to stomach ulcers, the spleen, enlarged liver, hepatic disorders, skin diseases, chest pain, cough, diabetes, rheumatism, and blood purification [21]. In addition, *Curcuma longa*, a species of this genus, has been shown to have anti-inflammatory properties [22]. Potent cytotoxic activity in a leukemic cell line, hemolysis, and antioxidant activity have also been evaluated in *Curcuma amada* [23]. Moreover, in vitro analysis of volatile oils that are obtained from *Curcuma caesia* leaf has been shown to have extensive anti-inflammatory, antioxidant, and antibiotic effects [24,25]. Furthermore, most of the species of this genus contain bioactive components, such as flavonoids, terpenes (above 40 monoterpenes and sesquiterpenes), phenolic compounds, and antioxidants [26,27,28]. In several countries in Asia, different components of these plant species have been utilized, either cooked or eaten as vegetables [29]. Due to possessing a good amount of starch, vitamins, grains, proteins, minerals, fats, etc., they are often viewed as nutrition-rich foods [30,31].

## 3. Anti-inflammatory Activity of the Genus *Curcuma*

The genus *Curcuma* of the family Zingiberaceae has been important for its anti-inflammatory activity since prehistoric times. Although hundreds or more species of *Curcuma* have been identified up until now, very few species have undergone the scientific investigation for anti-inflammatory actions described as follows.

### 3.1. Curcuma longa L.

*Curcuma longa* L., also known as turmeric, is used as a spice in our daily dishes and is the representative species of the genus *Curcuma*. Many studies regarding anti-inflammatory effects on this species have been conducted by scientists and researchers for decades [32]. Here, we summarize these articles and have found a vast range of anti-inflammatory properties of it, either on mice models or others that are exposed to different natural or synthetic inflammatory agents compared to several NSAIDs (Table 1 and Table 2).

Previously, the medicinal use of turmeric has been demonstrated using in vivo studies, in which turmeric was administered in the form of a gel in an inflamed rat model [32,33]. While working with turmeric oil, another study using a murine model in which inflammation was acutely induced by carrageenan and dextran and chronically by formalin reported a significant reduction in paw thickness in comparison with the standard drug used in the study [32].

Different extracts of *C. longa* have been investigated upon administration in study models (Table 1). In one case, collagen-induced arthritic rats were treated with a *C. longa* extract that arrested the degenerative changes in the bone and joints [34]. Potent action was shown to occur using aqueous extracts of *C. longa* and *B. aristata* against endotoxin-induced uveitis in rabbits when administered topically [34]. An oil-free aqueous extract (COFAE) of *C. longa* and curcuminoids showed significant anti-inflammatory effects against acute and chronic inflammation [35]. A recent study investigated the aerial parts of this species, where its extracts were applied on RAW264.7 macrophage cells. An *n*-butanol fraction significantly curbed the lipopolysaccharide-mediated induction of several mediators, such as nitric oxide, prostaglandin E_2_, and pro-inflammatory cytokines, by reducing the levels of nitric oxide synthase and cyclooxygenase-2 in a dose-dependent manner [36]. A polysaccharide fraction (F1) of a *C. longa* aqueous-based extract (NR-INF-02) was also investigated in classical rodent models of inflammation. F1 has been considered for its potential in acute (carrageenan-induced paw edema and xylene-induced ear edema) and chronic (cotton-pellet-induced granuloma) models of inflammation [37]. Dimethyl sulfoxide (DMSO) extracts and curcumin was investigated on human intervertebral disc cells and it was suggested that the intra-distal injection of curcumin could be an alternative approach to treating inflammation [38].

Several studies have compared *C. longa* with other species of different genera regarding its anti-inflammatory effect. For example, a study compared the anti-inflammatory activity of rhizomes of *C. longa* (turmeric), and *Zingiber officinale* (ginger) in rat adjuvant-induced arthritis (AIA) [39] and showed the anti-inflammatory activity of turmeric over ginger and indomethacin against the rheumatoid arthritis onset/progression displayed in AIA. Another study found that UP1304, a combination containing the standardized extract of *C. longa* rhizome and *Morus alba* root, was used to treat symptoms of arthritis [40].

**Figure 1:**
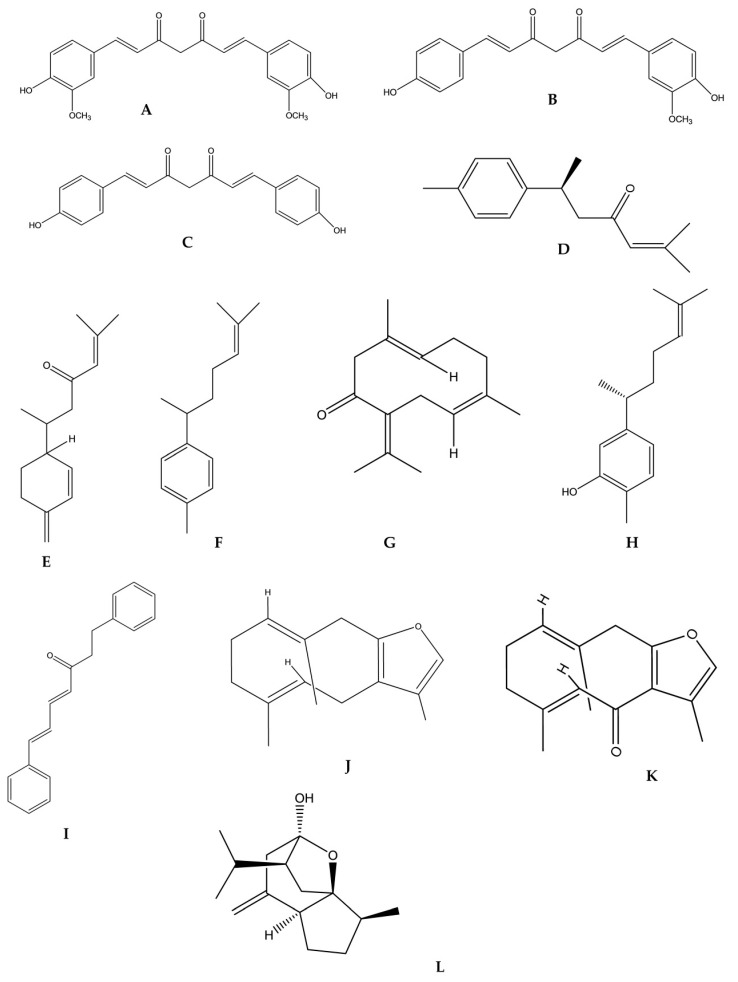
Structure of the (**A**) curcumin, (**B**) demethoxycurcumin, (**C**) bisdemethoxycurcumin, (**D**) ar-turmerone, (**E**) curlone, and (**F**) curcumene from *Curcuma longa*; structure of (**G**) germacrone, (**H**) xanthorrhizol, and (**I**) alnustone from *Curcuma xanthorrhiza*; structure of (**J**) furanodiene and (**K**) furanodienone from *Curcuma zedoaria*; structure of (**L**) curcumol from *Curcuma wenyujin*.

A few articles that include research and review are more specific in that these studies directly relate different constituents extracted from *C. longa* and their anti-inflammatory actions (Table 2). Curcumin is one of the most important compounds extracted from rhizomes of *C. longa* and was shown to be an anti-inflammatory agent in inflammatory bowel disease, pancreatitis, arthritis, chronic anterior uveitis, etc. [41]. It inhibits arachidonic acid metabolism, cyclooxygenase (COX), lipoxygenase (LOX), cytokines (interleukins and tumor necrosis factor), nuclear factor κB, and discharge of steroidal hormones, and might stabilize the lysosomal membrane and cause detaching of oxidative phosphorylation with high oxygen radical scavenging activity [41]. Another review article summarized the safety and anti-inflammatory activity of curcumin and supported the above mechanisms regarding providing anti-inflammatory actions [34]. *Helicobacter*-*pylori*-infected gastritis patients underwent investigations for the effectiveness of curcumin, but alone it may have limited the anti-bactericidal effect on *H. pylori* and the production of inflammatory cytokines [42]. Curcumin I, curcumin II (monodemethoxycurcumin), and curcumin III (bis-demethoxycurcumin) (Figure 1) from *C. longa* have demonstrated good inhibition of the COX-II enzyme [34].

A surprising piece of information is that the side effects of certain drugs can be minimized using curcuminoids from *C. longa*; 5-fluorouracil (5-FU)-induced intestinal mucositis in a murine model was treated with a mucoadhesive formulation containing curcuminoids (MFC) that displayed the anti-inflammatory effects of curcuminoids [50]. The structures of curcumin, demethoxycurcumin, bisdemethoxycurcumin, ar-turmerone, curlone, and curcumene are shown in Figure 1.

### 3.2. Curcuma xanthorrhiza Roxb.

Like *C. longa* Roxb., the *Curcuma xanthorrhiza* Roxb. rhizome is the most important part that exerts anti-inflammatory effects and xanthorrhizol (Figure 1) is the common constituent identified from the extracts responsible for anti-inflammatory actions.

The methanol extract of the rhizome of *C. xanthorrhiza* and its active constituent germacrone (Figure 1) have demonstrated anti-inflammatory effects [52]. In high-fat diet (HFD)-induced obese mice, the anti-inflammatory action of xanthorrhizol (XAN) and a *C. xanthorrhiza* extract (CXE) was investigated in a study with standardized XAN on inflammatory markers, where XAN and CXE significantly inhibited the production of inflammatory cytokines, such as TNF-α, IL-6, IL-1β, and C-reactive protein (CRP) in adipose tissue, liver, and muscle [53].

Three non-phenolic diarylheptanoids from *C. xanthorrhiza*, namely, *trans*,*trans*-1,7-diphenyl-1,3-heptadien-4-one (alnustone) (Figure 1), *trans*-1,7-diphenyl-1-hepten-5-ol, and *trans*,*trans*-1,7-diphenyl-1,3-heptadien-5-ol have demonstrated potential anti-inflammatory activity in carrageenan-induced hind paw edema in rats [54,55], where the non-phenolic linear 1,7-diarylheptanoids were proposed to represent a novel class of topical anti-inflammatory agents (Table 2) [56].

### 3.3. Curcuma zedoaria (Christm.) Roscoe

*Curcuma zedoaria* Christm., also known as white turmeric, was investigated for its anti-inflammatory actions and several published studies are available on it. Different extracts and their constituents show their distinct anti-inflammatory actions (Table 1 and Table 2).

One of the studies demonstrated the significant (*p* < 0.001) anti-inflammatory effects of all extracts from it, except methanol extract, in albino rats that were given carrageenan and histamine, resulting in hind paw edema. The effects were compared to a 10 mg/kg indomethacin intraperitoneal injection (i.p.) and 200 mg/kg of rumalaya forte, but amongst these extracts, 200 mg/kg of petroleum ether and 400 mg/kg of chloroform extracts exerted the greatest anti-inflammatory activity during the second to sixth hours [43]. In addition, the ethanolic root extract of *C. zedoaria* has also shown significant anti-inflammatory activity against the same study model [44]. The ethanolic rhizome extract of *C. zedoaria* possesses significant anti-inflammatory activity against a carrageenan-induced inflammatory response in rats in a concentration-dependent manner. Furthermore, the plant may possess one or more secondary metabolite(s) that have central and peripheral analgesic and anti-inflammatory activity [57].

Sesquiterpene from the methanolic extract of the rhizome of *C. zedoaria* was investigated using TPA-induced inflammation of mouse ears, where furanodiene and furanodienone (Figure 1J,K) suppressed the TPA-induced inflammation of mouse ears by 75% and 53%, respectively, at a dose of 1.0 μmol, which are effects that are comparable to that of indomethacin [58].

### 3.4. Curcuma phaeocaulis Val.

There are very few studies regarding this species that describe the anti-inflammatory effects. A study compared six species of *Curcuma* drugs, including *Curcuma phaeocaulis* Val., in adjuvant arthritis model mice. When the *C. phaeocaulis* methanol extract was administered orally one day before and after the adjuvant injection, paw swelling and serum haptoglobin levels were significantly inhibited, while curcuminoids were missing [45]. Another study about the anti-inflammatory effects of the ethanol extract of *C*. *phaeocaulis* and its fractions showed that the ethanol extract from it had some anti-inflammatory effects at certain concentrations but its three sub-fractions had only limited anti-inflammatory action [46].

### 3.5. Curcuma wenyujin Y.H.

*Curcuma wenyujin* Y.H. has undergone limited investigation for anti-inflammatory action, where one article depicted the aqueous extract of it in mice using different methods and finally ensured that it decreased the level of different inflammatory cytokines (Table 1). Moreover, it suggested that this species could be used in pelvic inflammation for better anti-inflammatory effects [59]. Curcumolide from *C. wenyujin* may have therapeutic potential for treating inflammatory diseases by inhibiting NF-κB activation and pro-inflammatory mediator production [60] (Figure 1).

### 3.6. Curcuma mangga Val.

Different extracts and their fractions of *Curcuma mangga* Val. exhibit anti-inflammatory effects, as described in a few studies (Table 1 and Table 2). The use of carrageenan-induced rat paw edemas and croton-oil-induced mouse edemas in inflammatory models was investigated using *C. mangga* ethanol extract (CME) and its fractions, such as water, chloroform, ethyl acetate, and hexane fractions from rhizome. CME and its fractions, especially chloroform and hexane fractions, displayed significant anti-inflammatory activities [34]. Another study was conducted on the anti-inflammatory mechanisms of *C. mangga* extract and its compounds against nitric oxide (NO) and prostaglandin E2 (PGE2) release using RAW 264.7 cells. Their constituents were found to down-regulate the mRNA expressions of iNOS and COX-2 in a dose-dependent manner [61].

### 3.7. Curcuma amada Roxb.

The rhizome of *Curcuma amada* Roxb. was investigated in only one article found in this regard that exhibited the result of ethanol extract from it when administered in albino rats using acute carrageenan paw edema and a chronic granuloma pouch model (Table 1). The study concluded by showing anti-inflammatory activity in cases of acute and chronic administration in albino rats [47].

### 3.8. Curcuma aeruginosa Roxb.

The anti-inflammatory effects of *Curcuma aeruginosa* Roxb. have not been adequately investigated yet. Although one study was conducted using its rhizomes extracts of chloroform, methanol, and water using heat-induced pain in mice and carrageenan-induced edema in rats, the study ended with no anti-inflammatory effect from any extracts [48].

### 3.9. Curcuma aromatica Salisb.

The anti-inflammatory role of essential oils (EOs) derived from *Curcuma aromatica* Salisb. rhizomes from twelve places in China was investigated by Xiang [62]. Ear edema was caused by 12-*O*-tetradecanolphorbol-13-acetate in rats in their research. Different classes of mice underwent numerous essential oil therapies, where ibuprofen was used to positively regulate them. In general, both EOs demonstrated dose-dependent anti-inflammatory behavior between 20.56 and 61.34%; this was remarkably higher than ibuprofen (17.84–54.57%), which is recognized for its anti-inflammatory effects. Furthermore, tissue healing from inflammation following treatment with both EOs was shown through histological and immunohistochemical studies. The cytokine analysis found that the output of COX2 and TNF-α in the EO-treated groups was substantially decreased relative to the untreated community. Furthermore, extracts of *C. aromatica* rhizomes were documented to possess promising anti-inflammatory activity comparable to prednisolone when evaluated on carrageenan-induced inflamed mice [49]. However, it is not shocking that *C. aromatica* extracts and essential oil possess a more potent anti-inflammatory activity than traditional pharmaceutical drugs since they produce and can have the stimulating impact of numerous powerful anti-inflammatory compounds, such 1,8-cineole, ar-turmerone, xanthorrhizole, borneol, curcumin, curdione, and linalool [48,49,63,64].

## 4. Inflammation and Natural Bioactive Compounds

Inflammation is already regarded as a significant phenomenon in cellular and sub-cellular diseases. This process comprises few cells involved in enhancing the production of pro-inflammatory chemical mediators (IL, TNF-α, NO, and PGs). The overproduction of mediators depends on the stimulus and tissue injury [65].

Phenolic compounds act in the same manner as NSAIDs, which are capable of reducing the chemical mediators of inflammation [66]. Here, dietary phenolics are connected to the inflammatory process and are associated with diseases that become potential targets to prevent these conditions [67]. The pathophysiology of certain forms of human cancer and inflammatory disorders was linked to the improper production of COX-2 and NO synthesis [68]. The curcumin from *C. longa* was reported to reduce the IL-1β, IL-6, IL-8, MMP1, MMP3, MMP13 and TNF-α in human intervertebral disc cells [38]. In addition, UP1304 and curcumin I–III suppress the enzymatic action of COX and LOX [34,40]. A similar result was found when using petroleum ether, which is an alcohol extract of curcumin that reduces the metabolism of arachidonic acid and inhibits the production of COX, LOX, cytokines (ILs and TNF), and NF-κB [42].

A study that was particularly focused on the assessment of the effect of xanthorrhizol from *C. xanthorrhiza* was conducted in a mice model exhibited significant inhibition of TNF-α, IL-6, and IL-1β in adipose tissue, liver, and muscle. The isolated phenolic compound curcumol found in *C. wenyujin* was reported to inhibit the production of the TNF-α and IL-6 [59], while curcumolide, a novel terpenoid, suppresses the lipopolysaccharide (LPS)-treatment-induced activation of NF-κB and inhibits the production of TNF-α, IL-6, IL-1β, NO, and reactive oxygen species (ROS) [60].

The extracts of the *C. zedoaria* rhizome have been reported to inhibit carrageenan-induced pro-inflammatory responses and suppress the acute inflammatory response induced by tetradecanoylphorbol-13-acetate (TPA) in rat and mouse ears [43,44]. Furthermore, the isolated compound germacrone from the methanol extract of *C. xanthorrhiza* rhizome demonstrated the inhibition of paw edema [52], whereas the non-phenolic diarylheptanoids displayed the significant inhibition of carrageenan-induced hind paw edema in rats [54,55].

**Figure 2:**
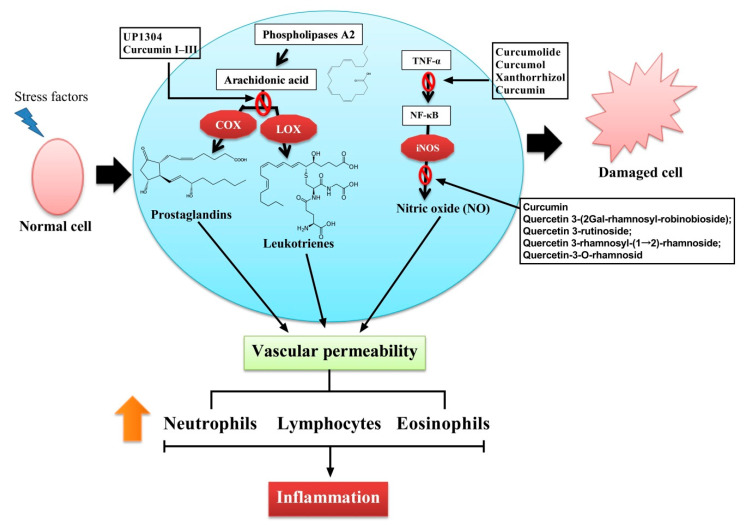
Anti-inflammatory activities of natural bioactive compounds.

Overall, most studies on the anti-inflammatory effects of phenolic compounds focus on plant extracts and isolated phenolic compounds. The studies on different extracts and their fractions of *C. longa* are rich in phenolic compounds, which displayed significant suppression of lipopolysaccharide-mediated induction of nitric oxide, prostaglandin E_2_, and pro-inflammatory cytokines by reducing the levels of nitric oxide synthase and cyclooxygenase-2 in a concentration-dependent manner in RAW264.7 macrophage cells [36]. Furthermore, *C. mangga* rhizome extracts and their fractions, namely, demethoxycurcumin, 15,16-bisnorlabda-8(17), 11-dien-13-one, (E)-15,15-diethoxylabda-8(17), 12-dien-16-al, and bisdemethoxycurcumin, displayed positive effect against inflammation. In this study, the result clearly revealed a decreased regulation of mRNA expression in inducible nitric oxide synthase (iNOS) in RAW 264.7 macrophage cells. Additionally, the extract and fraction of the *C. mangga* rhizome exhibited a decrease in the production of COX-2 in a dose-dependent manner [61]. The phenolic compounds known as curcuminoids display antioxidant activity that helps to inhibit the production of ROS, resulting in anti-inflammatory properties because of the blockade of COX, LOX, NO synthesis, and other inflammatory enzymes, and also disrupts the transduction of cellular signals through different mechanisms, including protein kinase C inhibition. Recent scientific evidence on the impact of curcuminoids on their anti-inflammatory effect using in vitro and in vivo studies is shown in Figure 2.

## 5. Commercial Uses of the Genus *Curcuma*

The species belonging to the *Curcuma* genus have been well-established in terms of their commercial values, such as food and medicinal values. Sixteen species possess high commercial values, and their rhizomes were assessed as the most important edible portion of the plants. A substantial amount of yellow dye originates from the rhizome of *Curcuma*, which has traditionally been used as food preservatives, flavoring agents, spices, and as a household cure for treating many diseases [69]. The rhizomes of *C. angustifolia*, *C. leucorrhiza*, and *C. caulina* are reasonable sources of carbohydrates and are also used as nutritious food substitutes, along with being a substitution for true arrowroot powder. In contrast, the rhizomes of *C. aeruginosa*, *C. amada*, *C. longa*, *C. aromatica*, *C. pierreana*, *C. xanthorrhiza*, *C. pseudomontana*, and *C. zedoaria* are often used as spices and dyes, as well as coloring and flavoring agents in food preparations, which is primarily due to their exotic fragrance. Additional sections, including tuberous roots, inflorescence, and rootstocks, are similarly good sources of carbohydrates and proteins and are used in cooking preparations and as food appetizers and vegetables [69,70,71]. In addition to food values, the species of the genus *Curcuma* possesses plenty of pharmacological activities, which make them valuable constituents in pharmaceutical industries. Mujumdar et al. found that the alcohol extracts derived from *C. amada* comprise several constituents, such as the functional groups of olefin, hydroxyl, carbonyl, and ester, which were found to have anti-inflammatory effects at acute and chronic administrations in albino rats [47]. Furthermore, rhizomes and other plant parts of *C. aeruginosa*, *C. caesia*, *C. angustifolia*, *C. phaeocaulis*, *C. leucorrhiza*, *C. mangga*, *C. longa*, and *C. purpurascens* have been established as a treatment for quite a lot of physiological disorders, such as dysentery, indigestion, stomach ulcer, gastrointestinal disorders, diabetes, enlarged liver and spleen, fever, boils, cough, scabies, body pain, chest pain, hepatic disorders, bruises, anorexia, rheumatism, dyspepsia, wound healing, sinusitis, bleeding, and infection [21,69,72,73,74].

## 6. Clinical and Preclinical Studies of Bioactive Compounds of *Curcuma* Species Based on the Anti-inflammatory Properties

The bioactive compounds of *Curcuma* species have been well-documented in clinical and preclinical studies for their anti-inflammatory activities. Curcumin is a major constituent of *C. longa* and its therapeutic potential against numerous diseases has been reported. A dosage of 50 mg/kg of curcumin for ten days before inducing colitis with 1,4,6-trinitrobenzene sulphonic acid contributed to substantial improvements in diarrhea, produced an amended colonic architecture, and caused a significant reduction in lipid peroxidation and neutrophil penetration. Decreased levels of NO and O_2_ radicals, as well as suppressed activation of NF-κB in the colonic mucosa, reduced inflammation, and improved symptoms, have also been documented [75]. Atoskar et al. investigated the impact of curcumin on spermatic cord edema relative to placebo and phenylbutazone after an operation for inguinal hernia or hydrocele. Here, 45 patients were regularly administered three times for 6 days following treatment, where group A was given 400 mg curcumin, group B was given 250 mg lactose powder placebo, and group C was given 100 mg phenylbutazone. The severity score dropped by 84.2%, 61.8%, and 86% in groups A, B, and C, respectively, by day 6. While on day 6, the severity scores for phenylbutazone and curcumin were identical, curcumin was demonstrated to be superior by decreasing all four inflammation parameters [76,77]. In addition, Kim et al. tested the anti-inflammatory and antihyperglycemic effects of xanthorrhizol and *C. xanthorrhiza* extract with consistent xanthorrhizol on (HFD)-induced obese mice. Xanthorrhizol, at dosage levels of 10 mg/kg/day or 25 mg/kg/day, greatly reduced the glucose levels of blood, as well as inhibited the development of inflammatory cytokines, such as TNF-α, IL-1β, IL-6, etc., in HFD-induced obese mice [53]. Chen et al. examined the anti-inflammatory properties of curcumol (bioactive compound of *C. wenyujin*) in lipopolysaccharide-induced RAW264.7 cells. Throughout this study, the authors investigated how curcumol obstructs lipopolysaccharide-induced nitric oxide production at concentrations ranging from 12.5–200 µM via the suppression of iNOS mRNA expression and the protein level. In addition, curcumol interrupts the LPS-induced development of TNF-α, IL-1β, and IL-6, both during the transcriptional and translational stages, and primarily works by disrupting JNK-mediated AP-1 instead of NF-κB. These findings include experimental molecular confirmation that curcumol, owing to its inhibitory role in the synthesis of multiple inflammatory drugs, could be a potential leading compound for a novel anti-inflammatory medication [78].

Furthermore, curcumin shows very poor bioavailability, as many studies show very low, or even undetectable, blood and extraintestinal tissue concentrations. The key reasons given for this are its poor absorption, fast metabolism, chemical instability, and rapid systemic elimination [79]. Clinical phase I trials showed that curcumin is safe in humans, even at a dose of 12 g per day [80]. Numerous methods have been used to improve curcumin bioavailability. These methods include [80,81]: Use in combination with other compounds.

1. Use as liposomal curcumin via liposomal technology.
2. Curcumin as nanoparticles.
3. Use as a curcumin–phospholipid complex.
4. New formulation (micelles, exosomes) to increase the bioavailability.

## 7. Conclusions and Future Perspectives

The present review summarizes the anti-inflammatory properties of the genus *Curcuma*. Based on the published literature, we can conclude with the following remarks. At first, it was observed that only a few species have been scientifically investigated among more than 100 species, where there is not enough published literature for each species, except *Curcuma longa*. Therefore, more scientific investigations are required on every species for their anti-inflammatory effects. Second, except for the roots of *Curcuma zedoaria* only rhizomes have been the subject of scientific investigations. These rhizomes have shown significant anti-inflammatory effects; therefore, scientific investigations on other plant parts may provide evidence for promising anti-inflammatory effects. Third, many published scientific articles showed the anti-inflammatory effects of *Curcuma longa* and others in rats and mice. The maceration extraction techniques were followed in the present *Curcuma* studies, while a comparative study of the extract effect is required using other extraction techniques, such as Soxhlet, injection, percolation, and hot extraction. Therefore, further investigations are required in this case. Finally, more scientific research and investigations are required to isolate single entities and constituents that are responsible for specific anti-inflammatory actions.

All nine edible *Curcuma* species mentioned in this review are critical because they possess culinary and medicinal properties, and thus, have great potential for use as functional foods and medicines. Studies related to consumer safety, clinical trials, in vivo investigations, and cytotoxic studies will be beneficial as information related to this area is limited. Additional research on the nutritional values, along with pharmacological studies of new uninvestigated compounds, is desirable and will provide immense opportunities for the development of new plant-based food and pharmaceutical products. Therefore, it is concluded that the genus *Curcuma* is a significant source of anti-inflammatory agents, justifying its traditional uses, but this genus has not undergone enough scientific investigations yet. However, scientists should consider this genus to develop alternative and complementary anti-inflammatory agents with its dosage formulations and other specifications. Moreover, regular consumption of alternative and complementary herbal products may become an effective and safe strategy to treat chronic inflammatory diseases.

More phytochemical analyses should be performed to identify more potent bioactive compounds from these plants. From these single chemical entities, it is possible to perform quantitative structural activity analysis to find out the potency of the fraction leading to the synthesis of more active compounds for drug development. Alternatively, through a systematic approach, one can prepare a reproducible and stable active extract for the development of a patentable product without adverse effects. Furthermore, the bioavailability and pharmacokinetics of these active extracts should be further explored to correlate the in vitro effects with in vivo efficacy and set a basis for clinical studies. In conclusion, the genus *Curcuma* is a treasure waiting for researchers interested in the development of safe and effective anti-inflammatory agents. Further studies on this genus will extend their clinical applications. The combined use of natural phytochemicals identified with other anti-inflammatory agents will open a new area of research.

## Figures and Tables

**Table 1:** Anti-inflammatory effects of various crude extracts from different *Curcuma* species.

Species of *Curcuma*	Plant Parts	Extracts/Fractions/Gels	Dose/Concentration	Assay	Study Model	Mechanism of Action	Ref.
*Curcuma longa*	Rhizome	Aqueous	0.1%	1	Rabbits	Significant reduction in the levels of inflammatory cells, proteins, and TNF-α in aqueous humor, thus reducing the severity of clinical signs and histopathologic changes of uveitis	[34]
	Rhizome	Oil-free aqueous	Three doses (20 mg/kg, 60 mg/kg, 180 mg/kg)	2, 3	Albino Swiss mice and Wistar rats	Significant reduction in ear weight and decrease in wet as well as dry weights of cotton pellets in both models	[35]
	Rhizome	Administered topically in gel form	3.33–33.3% of turmeric	4	Rats	Significant and dose-dependent inhibition of rat paw edema	[33]
	Rhizome	Polysaccharide fraction (F1) of an aqueous-based extract (NR-INF-02)	11.25, 22.5, and 45 mg/kg	2, 3, 4,	Rodent	Significant inhibition of paw and ear edema and reduction of wet and dry weights of cotton pellets	[37]
*Curcuma zedoaria*	Root	Petroleum ether, chloroform, and methanol	Petroleum ether, 200 and 400 mg/kg chloroform extracts	4, 5	Albino rats	n.m.	[43]
	Root	Ethanol	Ethanol at 200 and 400 mg/kg root extracts	4, 5	Rats	n.m.	[44]
*Curcuma phaeocaulis*	Rhizome	Methanol	500 mg/kg	6	Adjuvant arthritis model mice	Reduction in serum haptoglobin concentration	[45]
	Rhizome	Ethanol	10 to 80 μg/mL	7	Mouse RAW264.7 cells	Inhibition of nitrite production in inflammatory reactions	[46]
*Curcuma mangga*	Rhizome	Ethanol extract and its aqueous fractions, chloroform, ethyl acetate, hexane	200 mg/kg	4, 8	Rat, mouse	n.m.	[34]
*Curcuma amada*	Rhizome	Ethanol	40–80 mg/kg	3, 4	Albino rats	n.m.	[47]
*Curcuma aeruginosa*	Rhizome	Chloroform, methanol, and water	3.03 g/kg	4	Rats	No anti-inflammatory effect	[48]
*Curcuma aromatica*	Rhizome	Ethanol	1–2 g	9	Albino mice	n.m.	[49]

n.m.—not mentioned, 1—endotoxin-induced uveitis, 2—xylene-induced ear edema, 3—cotton pellet-induced granuloma, 4—carrageenan-induced paw edema, 5—histamine-induced hind paw edema, 6—adjuvant-induced paw swelling, 7—in vitro test in mouse macrophage cell line RAW264.7 cells, 8—croton oil-induced ear edema, and 9—arachidonic acid-induced ear inflammation.

**Table 2:** Anti-inflammatory activity of bioactive compounds isolated from different *Curcuma* species.

Species	Plant Parts	Bioactive Compounds	Extracts/ Fractions/Oils	Assay	Study Model	Results/Mechanism of Action	Ref.
*Curcuma longa*	Aerial parts	Quercetin 3-(2Gal-rhamnosyl-robinobioside), quercetin 3-rutinoside, quercetin 3-rhamnosyl-(1→2)-rhamnoside, and quercetin-3-O-rhamnoside	Methanol (CH_2_Cl_2_ and n-BuOH fractions)	1	RAW264.7 macrophage cells	Significantly suppressed the lipopolysaccharide-mediated induction of nitric oxide, prostaglandin E_2_, and pro-inflammatory cytokines; reduced the levels of nitric oxide synthase and cyclooxygenase-2 in a concentration-dependent manner	[36]
	n.m.	Curcumin	DMSO	2	Human intervertebral disc cells	Reduced levels of IL-1β, IL-6, IL-8, MMP1, MMP3, and MMP13, and up-regulated TNF-α	[38]
	Rhizome and root barks	UP1304 (10% curcumin and 2% mulberroside)	Ethanol	3	Rat	Inhibition of the enzymatic activities of COX and LOX	[39]
	Rhizome	Curcumin	Petroleum ether, alcohol	4, 5, 6		Inhibition of AA metabolism, COX, LOX, cytokines (ILs and TNF), and NF-κB	[42]
	Rhizome	Curcuminoids	Mucoadhesive formulation containing curcuminoids (MFC)	7	Adult Swiss male mice	Stimulated cell proliferation by ≈90% in the epithelial cells’ lining from villi and crypts, and reduced MPO levels and MDA formation by 60% and 44%, respectively	[50]
	n.m.	Ar-turmerone (61.79%), curlone (12.48%), curcumene (6.11%), etc.	Essential oil (turmeric oil)	3, 8, 9	Mice	Significant reduction in paw thickness	[51]
	n.m.	Curcuminoids, bisdemethoxycurcumin (2.5 to 6.5%), demethoxycurcumin (15 to 25%), and curcumin (70 to 80%)	Turmeric extract (Sabinsa Company, Malaysia)	10	Rats	Arrested the degenerative changes in the bone and joints	[32]
	Rhizome	Phenolic curcuminoids: curcumin, demethoxycurcumin, and bisdemethoxycurcumin	n.m.	5	Rats	Significantly suppressed the incidence and severity of arthritis by increasing/decreasing the production of anti-inflammatory/pro-inflammatory cytokines, respectively	[39]
	Rhizome, roots	Curcumin	n.m.	11	Animal and human	Inhibition of phospholipase, LOX, COX-2, LTs, Tx, PGs, NO, collagenase, elastase, hyaluronidase, MCP-1, Interferon-inducible protein, TNF-α, and IL-12	[34]
	n.m.	Curcumin	n.m.	12	*H.-pylori*-infected gastritis patients	Limited anti-bactericidal effect on *H. pylori* and on the production of inflammatory cytokines	[42]
		Curcumin I, curcumin II (monodemethoxycurcumin), and curcumin III (bisdemethoxycurcumin)	n.m.		n.m.	Inhibition of lipid peroxidation COX-I and COX-II enzymes	[34]
		Curcumin (diferuloylmethane), demethoxycurcumin, and bisdemethoxycurcumin	Volatile oils (tumerone, atlantone, and zingiberone)	3, 9, 13	n.m.	Significant inhibition of paw edema and curcumin has potential as a therapeutic agent against these anti-inflammatory diseases or conditions	[41]
*Curcuma xanthorrhiza*	Ehizome	Germacrone	Methanol, ether-soluble fractions, n-hexane soluble fractions	3, 14	Rats, mice	Significantly inhibited edema, vascular permeability, and the number of writhes	[52]
	Rhizome	Xanthorrhizol	Ethanol	15	Mice	Significantly inhibited the production of inflammatory cytokines, such as TNF-α, IL-6, IL-1β, and C-reactive protein in adipose tissue, liver, and muscle	[53]
	Rhizome	*trans*,*trans*-1,7-Diphenyl-1,3-heptadien-4-one (alnustone), *trans*-1,7-diphenyl-1-hepten-5-ol, and *trans*,*trans*-1,7-diphenyl-1,3-heptadien-5-ol	Hexane	3	Rats	Significant anti-inflammatory activity	[54,55]
		Non-phenolic linear 1,7-diarylheptanoids	n.m.	16	Murine model	Potent inhibition of ethyl-phenylpropiolate-induced ear edema	[56]
*Curcuma zedoaria*	Rhizome	Tannins, saponins, flavonoids, gums, carbohydrates, steroids, alkaloids, reducing sugars, and terpenoids	Ethanol	3, 17	Long Evans rats	Significantly inhibited the carrageenan-induced inflammatory response in a dose-related manner and protein denaturation in vitro	[57]
	Rhizome	Sesquiterpene: furanodiene (J) and furanodienone (K)	Methanol	18	Mouse	Suppressed the TPA-induced inflammation of mouse ears	[58]
*Curcuma wenyujin*		Curcumol	Aqueous	3, 4, 14	Mice, rats	Decrease the levels of TNF-α and IL-6; significant anti-inflammatory effects against all the test and pelvic inflammation	[59]
		Curcumolide	n.m.	1	RAW 264.7 macrophages	Suppress LPS-induced NF-κB activation, and decreased TNF-α, IL-6, IL-1β, NO, and reactive oxygen species (ROS) production	[60]
*Curcuma mangga*	Rhizome	Demethoxycurcumin, 15,16 bisnorlabda-8(17), 11-dien-13-one, (E)-15,15-diethoxylabda-8(17),12-dien-16-al, bisdemethoxycurcumin	Chloroform fraction, n-hexane fraction	1	RAW 264.7 cells	Down-regulation of the mRNA expressions of iNOS and COX-2 in a dose-dependent manner	[61]
*Curcuma aromatica*	Leaves and rhizomes	Ar-Turmerone, borneol, curcumin, demethoxycurcumin, linalool, xanthorrhizol,	Ethanol	19	Albino mice	n.m.	[49]

n.m.—not mentioned; DMSO—dimethyl sulfoxide; 1—lipopolysaccharide-mediated induction of nitric oxide, prostaglandin E2, and pro-inflammatory cytokines; 2—IL-1β-induced anti-inflammatory/catabolic cascade; 3—carrageenan induced paw edema; 4—cotton pellet method; 5—adjuvant-induced arthritis; 6—inflammatory skin conditions and allergies; 7—5-fluorouracil (5-FU)-induced intestinal mucositis; 8—dextran induced acute inflammation; 9—formalin-induced chronic inflammation; 10—collagen-induced arthritis; 11—in vitro, animal, and human studies; 12—*H*.-*pylori*-infected gastric mucosa and peptic ulcer diseases; 13—ulcerative colitis, rheumatoid arthritis, pancreatitis, osteoarthritis, uveitis, inflammatory pseudotumors, dyspepsia, irritable bowel syndrome, and inflammatory bowel disease; 14—acetic-acid-induced vascular permeability and writhing symptoms; 15—high-fat-diet-induced obese mice; 16—ethyl-phenylpropiolate-induced ear edema; 17—in vitro protein denaturation method; 18—12-*O*-tetradecanoylphorbol-13-acetate (TPA)-induced inflammation of mouse ears to produce ear edema in mice; 19—arachidonic-acid-induced ear inflammation.

## Data Availability

Available data are presented in the manuscript.
